# Intestinal bacteria modulate the foraging behavior of the oriental fruit fly *Bactrocera dorsalis* (Diptera: Tephritidae)

**DOI:** 10.1371/journal.pone.0210109

**Published:** 2019-01-16

**Authors:** Mazarin Akami, Awawing A. Andongma, Chen Zhengzhong, Jiang Nan, Kanjana Khaeso, Edouard Jurkevitch, Chang-Ying Niu, Boaz Yuval

**Affiliations:** 1 Department of Plant Protection, College of Plant Science & Technology, Huazhong Agricultural University, Wuhan, China; 2 Department of Biological Sciences, Faculty of Science, University of Ngaoundere, Ngaoundere, Cameroon; 3 Department of Microbiology and Plant Diseases, The Robert H. Smith Faculty of Agriculture, Food and Environment, The Hebrew University of Jerusalem, Rehovot, Israel; 4 Department of Entomology, The Robert H. Smith Faculty of Agriculture, Food and Environment, The Hebrew University of Jerusalem, Rehovot, Israel; University of Thessaly School of Agricultural Sciences, GREECE

## Abstract

The gut microbiome of insects directly or indirectly affects the metabolism, immune status, sensory perception and feeding behavior of its host. Here, we examine the hypothesis that in the oriental fruit fly (*Bactrocera dorsalis*, Diptera: Tephritidae), the presence or absence of gut symbionts affects foraging behavior and nutrient ingestion. We offered protein-starved flies, symbiotic or aposymbiotic, a choice between diets containing all amino acids or only the non-essential ones. The different diets were presented in a foraging arena as drops that varied in their size and density, creating an imbalanced foraging environment. Suppressing the microbiome resulted in significant changes of the foraging behavior of both male and female flies. Aposymbiotic flies responded faster to the diets offered in experimental arenas, spent more time feeding, ingested more drops of food, and were constrained to feed on time-consuming patches (containing small drops of food), when these offered the full complement of amino acids. We discuss these results in the context of previous studies on the effect of the gut microbiome on host behavior, and suggest that these be extended to the life history dimension.

## Introduction

Animals forage for nutritional resources in order to satisfy their requirements for growth and reproduction [1,2]. This behavior is constrained by spatial and temporal factors (biotic and abiotic), and modulated to a large extent by the phenotypic plasticity and metabolic state of each organism [3]. Evidence from numerous studies suggests that insects (and other arthropods) are capable of tailoring their foraging activity and ingestion of nutrients in a manner that corresponds to their specific requirements [4] (and references therein).

In insects, responses to environmental stimuli are modulated by substrate specific chemoreceptors, whose sensitivity is modulated by the nutritional status of the individual [5–7]. Thus, for example, in the vinegar fly *Drosophila melanogaster* (Diptera: Drosophilidae), flies deprived of amino acids exhibit an enhanced response to amino acids missing from their diets [8]. Similarly, tephritid fruit fly sensory responses and foraging activity are affected by nutritional status [9–11].

The microbiome resident in the gut of arthropod (and vertebrate) hosts adds another layer of complexity to the modulation of behavior in general and foraging behavior in particular [12–14]. This effect has been demonstrated along the various steps of the nexus connecting the gut and its microbiome to behavior, through metabolism, the immune and nervous systems, and sensory receptors. Thus, in *D*. *melanogaster*, the microbiota has multiple impacts on metabolism such as immune homeostasis, lipid and carbohydrate storage and vitamin sequestration [15–17]. These effects are extended to responses to food and ultimately affect foraging activity. In *Tenebrio molitor* (Coleoptera: Tenebrionidae) mealworms, individuals whose immune system is activated by a pathogen consume significantly more proteinaceous food than healthy individuals [18]. Conversely, stinkbug (*Megacopta punctatissima*, Hemiptera: Plataspidae) nymphs that acquire symbionts after hatching exhibit lower activity levels than symbiont free nymphs [19]. Recently, Wong *et al*. [20] demonstrated that the microbiome of *D*. *melanogaster* influences the olfactory sensitivity and foraging behavior of hosts in a manner that apparently benefits the bacteria specifically. Remarkably, there is evidence that the nutritional status of the host interacts with the microbiome to control foraging behavior [21]. In *D*. *melanogaster*, the absence of specific amino acids will trigger specific appetites for the missing nutrient. However, the presence of bacteria (that presumably could provide the missing amino acid), overrides such preferences [22].

Tephritid fruit flies (Diptera: Tephritidae) harbour communities of bacteria dominated by species of Enterobacteriacae [23]. These microbes have been shown to be involved in Nitrogen fixation [24,25], reproductive success [26,27], temporal host range expansion [28], protection from pathogens [29] and detoxification [30].

Adult tephritid flies require a mixed diet consisting of carbohydrate and protein, or at least protein precursors [31]. These nutrients are acquired by active foraging during daylight hours [32]. Sugars are acquired from nectar, honeydew and fruit juices, while nitrogenous compounds are taken up by feeding on bird feces, or in some cases bacteria on the phylloplane [33]. The presence of gut bacteria in adult flies contributes to their nutrition, specifically by brokering intractable sources of Nitrogen into essential amino acids. Thus, *Bactrocera oleae* (Diptera: Tephritidae) females which were experimentally deprived of gut bacteria were unable to produce eggs when they only had access to non-essential amino acids [31,34]. Foraging for food is risky, as active flies are exposed to predators and incur a considerable investment of time and energy.

The oriental fruitfly *Bactrocera dorsalis* (Diptera: Tephritidae) is one of the most invasive, multivoltine and polyphagous members of the Tephritidae family. This fly causes considerable loss of cultivated crops in most western and eastern parts of Asia and attacks over 350 host species worldwide [24,35]. Studies based on Polymerase Chain Reaction-Denaturing Gradient Gel Electrophoresis (PCR-DGGE) fingerprinting and High throughput pyrosequencing of the 16S rRNA gene have highlighted the prevalence of microbial communities inhabiting the gut [24,36–38] and reproductive organs of this insect [39], which play critical roles in host physiology and reproduction [39,40]. Pyrosequencing analysis of the *B*. *dorsalis* microbiome reveals 172 Operational Taxonomic Units assigned to 6 phyla (with Firmicutes as the most abundant in adult stages), 5 families, and 13 genera [24,41]. The predominant bacterial family in most of the previous studies was Enterobacteriaceae from which many cultivable species were identified, such as *Klebsiella oxytoca*, *Enterobacter cloacae*, *Morganella sp*., *Moraxella proteus*, *Providencia rettgerii* and *Citrobacter freundii*. Other bacterial species like *Pseudomonas aeruginosa* (Pseudomonadaceae), *Enterococcus phoeniculicola* (Enterococcaceae), *Lactobacillus lactis* (Streptococcaceae) and the genus *Listeria* (Listeriaceae) were also identified from *B*. *dorsalis* [27,37,38].

In the present study we examine the hypothesis that in the Oriental fruit fly, the presence or absence of gut symbionts will affect foraging behavior and nutrient ingestion. We offered protein-starved flies, symbiotic or aposymbiotic, a choice between diets containing all amino acids or only the non-essential ones. The different diets were presented in a foraging arena as drops that varied in their size and density, creating an imbalanced foraging environment. We predicted that symbiotic flies would consistently choose the diet that was most profitable in terms of foraging time. Conversely, we predicted that flies lacking symbionts would be constrained to forage on diets containing all amino acids, while incurring costs of increased exposure and foraging time.

## Materials and methods

### Fly rearing and handling

*Bactrocera dorsalis* larvae were collected from infested fruits from the experimental orange orchard of Huazhong Agricultural University (30°4’N and 114°3’ E). The larvae were carefully removed after peeling the orange fruits and allowed to develop in a wheat-bran based larval artificial diet. The third instar larvae were allowed to pupate in sterile sand under laboratory conditions and the resulting adults were kept under rearing since 2014 [24]. At each generation, flies were reared as described by Nash and Chapman [42] with slight modifications. Briefly, 100 adult males and females were housed in 5L cages at equal proportions. These cages were maintained under controlled environment: 12:12 light-dark photoperiod; temperature 26±3°C, and 57±5% relative humidity. Water was provisioned *ad libitum* and the adult diet consisted of Tryptone (25 g/L), Yeast extract (90 g/L), Sucrose (120 g/L), Agar powder (7.5 g/L), Methyl-p-hydroxybenzoate (4 g/L), Cholesterol (2.3 g/L), Choline chlorite (1.8 g/L), Ascorbic acid (5.5 g/L) dissolved in 1 L of distilled Water and steamed at 55°C for 20 min. The larval diet consisted of the same ingredients of adult diet (as described above) to which we added 250 g/L of wheat bran.

### Symbiotic and aposymbiotic flies

Symbiotic and aposymbiotic flies were produced from the laboratory established colony. Until day 4 post-emergence, they were fed on sugar diet (Su) consisting of 60% sucrose and minerals, and then were divided in two groups of 30 flies each: the symbiotic flies (15 males and 15 females) were fed sugar diet (without antibiotics) till day 7 whereas the aposymbiotic ones were fed the same sugar meal but inoculated with antibiotics (3mcg/mL Norfloxacin and 5mcg/mL Ceftazedime) till day 7 [31]. The diets were provisioned in 9 cm petri dishes presented in sterilized cotton wool. The antibiotics were selected after *in vitro* susceptibility test to 7 bacterial isolates and their *in vivo* capacity to significantly clear the gut of *B*. *dorsalis* within four feeding days (Table 1). The 3mcg/mL and 5mcg/mL, respectively, represent the minimum inhibitory concentrations (MIC) of the selected antibiotics.

**Table 1:** Antibiotics susceptibility testing by disc diffusion of gut bacteria isolated from the oriental fruit fly *Bactrocera dorsalis*.

No.	Antimicrobial Agent	Disc potency (μg/piece)	*Enterococcus faecalis* DBS-LAZ-13/17 (MG231268)	*Klebsiella oxytoca* FQH (MF144436)	*Bacillus cereus* L90 (KC428751)	*Enterobacter cloacae* ATCC13047 (NR_102794.2)	*Pantoea dispersa* DSM30073(NR_116797)	*Proteus mirabilis* NCTC11938 (NR_043997)	*Staphylococcus sciuri* *IESE*:*STI (KU962123)*	
1	Ampicillin	10	R	I	S	I	R	R	R	
2	Ceftazidime	30	S	S	S	S	S	S	S	*
3	Chloramphenicol	30	S	S	I	R	S	I	I	
4	Ciprofloxacin	5	I	S	S	S	R	S	S	
5	Clindamycin	2	R	I	R	R	R	R	R	
6	Erythromycin	15	R	R	R	R	R	R	R	***
7	Gentamicin	10	S	S	R	S	S	S	S	**
8	Kanamycin	30	S	S	R	S	S	S	S	**
9	Minocyclin	30	S	S	R	R	I	I	R	
10	Norfloxacin	10	S	S	S	S	S	S	S	*
11	Ofloxacin	5	S	S	R	S	S	S	S	*
12	Penicillins	10	I	R	R	R	R	R	R	***
13	Piperacillin	100	S	S	S	I	R	S	R	
14	PolymyxinB	300	S	S	R	S	S	S	S	**
15	Tetracyclin	30	S	S	R	R	R	R	I	***

Our isolated bacteria sequences share 100% similarity with species from GenBank whose strains and accession numbers are provided.

**R** = Resistant, **S** = susceptible, **I** = intermediate. R, S and I were determined after the *in vitro* susceptibility test.

* = Antibiotics potent to all gut bacterial isolates

** = Antibiotics potent to all bacterial isolates except *Bacillus cereus*

*** = Non potent antibiotics

To confirm the aposymbiotic status of the flies, 15 flies from each group were separately removed, individually anaesthetized by keeping them at -20°C for 20 min and then dissected. Their individual guts were aseptically homogenized and the gut homogenates were serially diluted up to 10^−7^ dilution in deionized distilled water and 100 μl of each dilution was spread onto LB-Agar plates (pH 7.2–7.4) and incubated at 30°C for 48–78h. Then, the Colony Forming Units (CFU) resulting from the bacterial colonies on each plate were averaged and analyzed within and between samples. The estimation level of the cultivated microbial communities in antibiotic treated flies gave 1.694x10^2^±26.1 CFUs g^-1^.gut^-1^ (mean ± SE of 15 individual flies) representing just 0.054% of the total bacterial communities of the normal flies which was 3.127x10^6^±8.24x10^2^ CFUs g^-1^.gut^-1^ (Independent T-Test, F = 26.809; t = 14.517; df = 1, 28; P < 0.0001). After this estimation, the antibiotic treated flies were confirmed aposymbiotic and were used as such in subsequent experiment. All the flies were starved for 24h before experiments.

### Preparation of experimental diets

Three different diets were prepared. A full diet (F) containing all amino acids (essential and non-essential), sucrose, and minerals (Table 2), required for an optimal maintenance and reproductive development of adult flies [31]; a non-essential amino acid diet (NE) containing exclusively non-essential amino acids, sucrose and minerals. A Sugar diet (Su) was provided before the experiments. The diet ingredients and preparation procedures were done as described by Ben-Yosef *et al*. [31].

**Table 2:** Nutrient composition of the experimental diets.

Ingredients	Components	Contents (mg)	
		F	NE
**Essential amino acids**	L-arginine	50.45	
	L-histidine	21.54	
	L-isoleucine	26.64	
	L-leucine	51.02	
	L-lysine	27.78	
	L-methionine	13.04	
	L-phenylalanine	33.44	
	L-threonine	25.51	
	L-tryptophan	13.60	
	L-valine	37.41	
**Non-essential amino acids**	L-alanine	36.85	36.85
	L-aspartic acid	53.28	53.28
	L-aspartic acid	19.27	19.27
	L-glutamic acid	185.36	185.36
	Glycine	42.51	42.51
	L-proline	58.95	58.95
	L-serine	36.85	36.85
	L-tyrosine	22.67	22.67
	FeSO_4_	2.50	2.50
	MnSO_4_	0.63	0.63
	ZnCl2	0.63	0.63
**Minerals and salts**	CuSO_4_	0.31	0.31
	MgSO_4_	20.00	20.00
	KH_2_PO_4_	84.65	84.65
	Ca(H_2_PO_4_)2	10.00	10.00
	KCl	117.00	117.00
	NaCl	45.00	45.00
	White sugar	10000.0	10000.0
	DDW	50000.00	50000.00

F: Full diet; NE: Non-essential amino acid diets; DDW: Deionized distilled water.

### Experimental procedures

**Fig 1:**
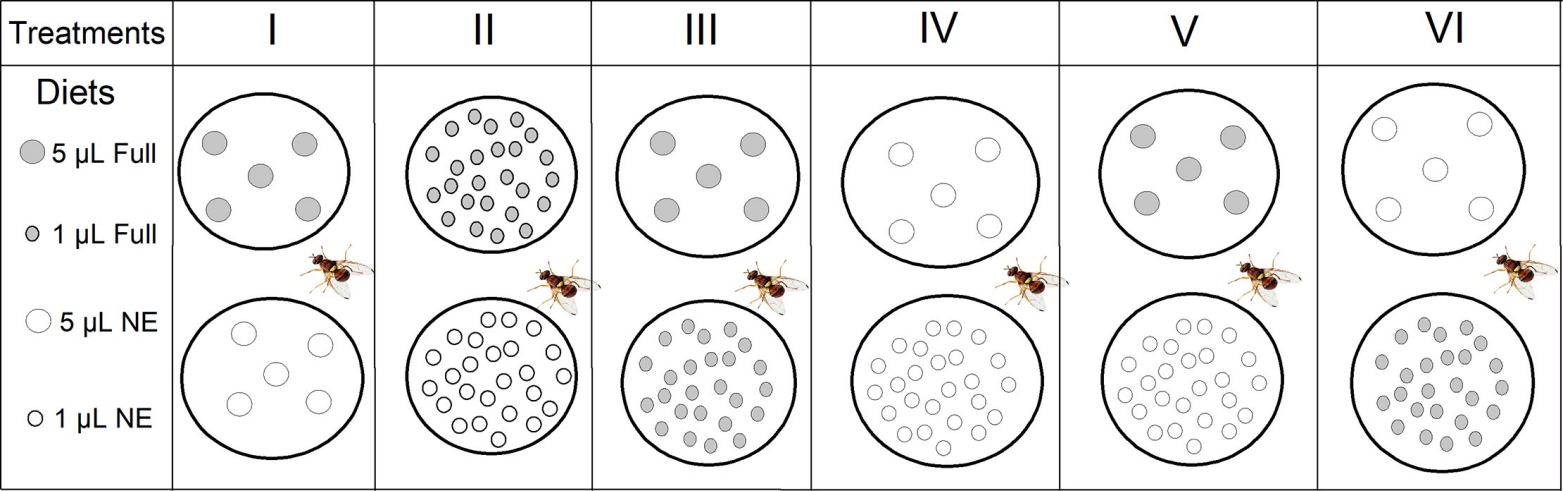
Experimental arenas of the effect of diet quality and density on foraging decisions by the oriental fruit fly *Bactrocera dorsalis*. Full diets contain all amino acids, and NE diets contain only the non-essential amino acids.

Following the seven day preparatory period during which flies were fed only sugar (as described above), an individual fly from each treatment was transferred to a 20 x 20 cm cage and allowed to acclimatize for 20 minutes before introducing a pair of petri dishes containing combinations of two different diet types (Full or NE) at different densities (Fig 1). To create different foraging environments, 25 drops of 1 μL volume (very small so as to force the flies to seek out many drops in order to become satiated), or 5 drops of 5 μL volume were pipetted onto each dish. Six treatment groups were set up representing six different foraging environments (Fig 1). Each of the six treatments was replicated 15 times (15 flies) and each replicate consisted of observing the protein starved individual male or female (symbiotic and aposymbiotic) for 1 hour. To motivate foraging behavior and allow the flies to recover from antibiotic effects, all the flies were starved for 24 hours before experimental trials. For each replicate (single fly in foraging environment), the response to the food drops (landing on a drop), Data on latency (duration from diet exposure to the initial landing), the number of flies that landed on a food drop (responses), the choice of diet made, the number of drops consumed (ingestion), and the time spent feeding were recorded and analyzed within and between treatments. Only drops that disappeared completely as a result of the fly feeding on it were considered in the estimation of the ingestion. In the rare cases when a food drop was partially consumed (n = 12 out of n = 360 feeding observations in symbiotic and aposymbiotic, male and female flies), the feeding event was discarded from the analyses.

### Statistical analyses

All data were tested for homogeneity of variances using Levene’s tests. To determine the important factors that shape the foraging behavior of experimental flies, variables of overall response (the number of flies that visited a food drop per treatment) and latency were analyzed (each one separately) using the ordinary least squares regression model (SPSS 20.0 software) with sex, symbiotic status, treatment and the interaction between symbiotic status and treatment as effects. Pearson Chi-Square test was used to determine the significance of responses based on the effects (treatments, sex and symbiotic status). Colony Forming Units of antibiotics treated and untreated samples were analyzed using an independent T-Test. The One-Way Analysis of Variance (ANOVA) was performed to analyze data on landing, the number of drops consumed, and the time spent and switching using SPSS 20.0 software (Statsoft Inc, Carey, NJ, USA). Tukey’s HSD Test (HSD) test was used for mean separations within and between each treatment. Differences among measured parameters were considered significant when the *p* values were less than 0.05 after comparison with HSD test. All results were expressed as the means with standard errors (SE), except data on the overall responses. OriginPro software version 8.5.1 was used to draw curves and graphs. All data collected and details on statististic models used can be found in S1 Table.

## Results

### Responses to the experimental arenas

**Fig 2:**
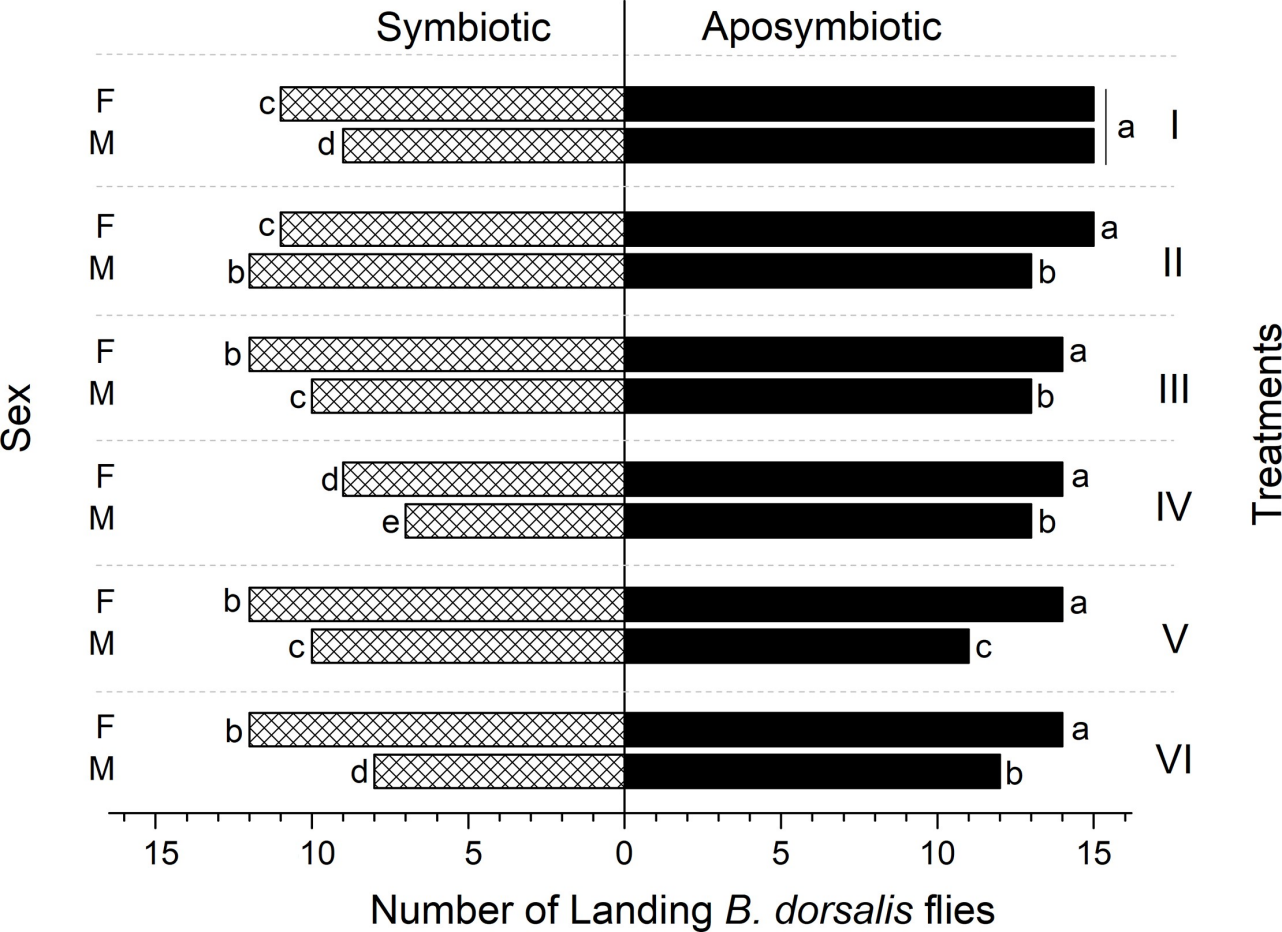
Response parameters of *Bactrocera dorsalis* females to experimental diets. Each response datum consisted of the overall number of flies which landed in different treatments regardless the diet quality or drop size.

Overall, of the 15 aposymbiotic flies in each treatment, on marginal average 14.83±0.37 females and 14.67±0.75 males, responded to the diets presented, while in symbiotic groups, only 10.83±1.34 females and 8.67±1.97 males responded out of 15 flies on average (Fig 2).

**Fig 3:**
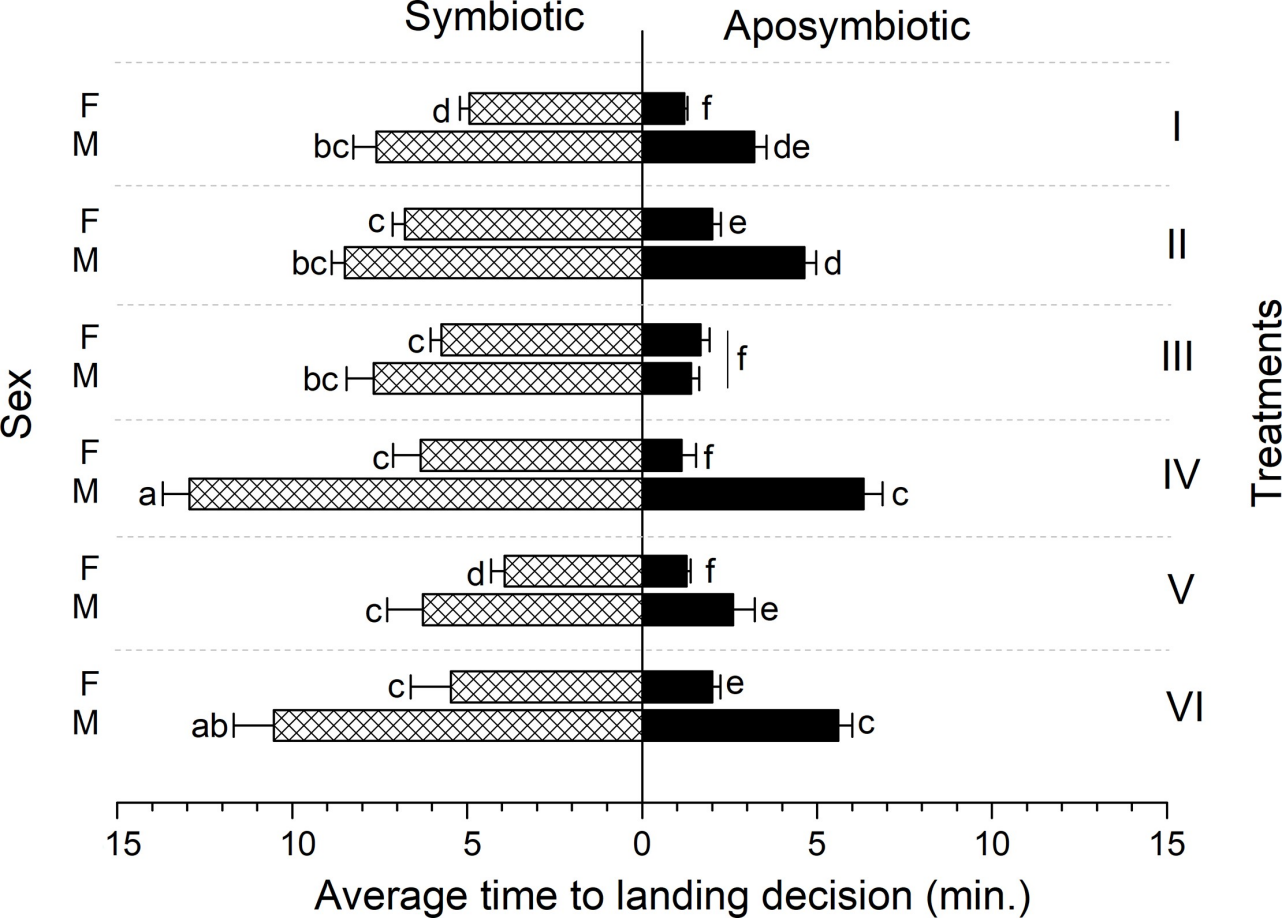
Response parameters of *Bactrocera dorsalis* males to experimental diets as affected by the symbiotic status, sex and treatments. Each latency datum is presented as a Mean±SE of the overall latency of responded flies in each treatment.

In general, most of the aposymbiotic flies landed. The overall response (number of responding flies per treatment) of symbiotic and aposymbiotic flies to the different treatments was significantly affected by symbiotic status (Ordinary Least Squares Regression Model, F = 15.834; df = 3; r^2^ = 0.839; t = 6.048; P < 0.001), and by sex (F = 15.83; df = 3; r^2^ = 0.839; t = -2.946; P = 0.043) (Fig 3). Pearson Chi-Square test revealed significant associations between the symbiotic status, sex and the responses (χ^2^ = 4.756, df = 1, P = 0.029), while no significant interaction exist between the treatments and the responses (χ^2^ = 8.621, df = 5, P = 0.125).

The treatment itself did not affect the overall response of flies (F = 15.834; df = 3; r^2^ = 0.839; t = -1.498; P = 0.197), but significantly influenced the latency to respond by either reducing or delaying the time elapsed to the first landing decision (F = 11.834; df = 3; r^2^ = 0.796; t = 0.216; P < 0.0001) (Fig 3). The latency to respond in the experimental chambers was similarly affected by symbiotic status (F = 11.538; df = 3; r^2^ = 0.796; t = -4.929; P < 0.0001) and sex (F = 11.538; df = 3; r^2^ = 0.796; t = 3.24; P = 0.04) (Fig 3). In general, aposymbiotic flies responded faster in the experimental chambers than the symbiotic ones, and females in aposymbiotic groups landed on food drops faster than the males (Fig 3).

### Switching behavior

Shifting from one diet to another was common, and showed a clear trend. Switching from Full to NE diets was not affected by sex (Linear Regression, F = 0.857, df = 1, P = 0.373), but was significantly affected by the symbiotic status (Linear Regression, F = 30.857, df = 1, P < 0.0001) while the sex and symbiotic status did not affect the switching of flies from NE to Full diets, respectively (Linear Regression, F = 1.829, df = 1, P = 0.201 and F = 1.829, df = 1, P = 0.433, respectively) (Table 3). No aposymbiotic flies which landed on the Full diet shifted to the NE diet and those which initially landed on the NE diet recorded a faster shifting latency (time to move from an initial patch to another) toward the Full diet in comparison with the symbiotic females and males (F = 10.857; df = 3,12; P = 0.001, and F = 10.857, df = 3,12; P = 0.012, respectively) (Table 3). However, 1 symbiotic female and male among those that initially landed on the Full diet shifted to the NE diet, but within a long shifting latency (F = 15.62, df = 3, 12; P = 0.001 and F = 15.62, P = 0.008, in females and males, respectively) (Table 3).

**Table 3:** Switching behavior of *B*. *dorsalis* as influenced by diet quality. The analysis involved only treatments with different diet types regardless the drop size. Each value is presented as a Mean ± SE of the four treatments, each consisting of 15 replications.

Initial landing	Shifting to	Symbiotic		Aposymbiotic	
		Females	Males	Females	Males
Full diet	NE diets	1.25±0.48a	1.75±0.25a	0.00b	0.00b
	Latency (min.)	28.25±1.8A	19±2.74B	0.00C	0.00C
NE diet	Full diets	2±0.41a	2.25±0.25a	1.25±0.48a	2.25±0.63a
	Latency (min.)	15.75±1.11A	12±0.08A	5.75±1.65B	5±1.29B

Means of shifting flies and shifting latency followed by different letters (small and capital letters, respectively) within rows are statistically different after Tukey HSD Test at P = 0.05.

### Ingestion

Overall, the number of drops consumed depended significantly on drop size, diet (full or NE), treatment (foraging environment), sex and symbiotic status of the flies observed (ANOVA; F = 45.86, df = 5, P < 0.0001, R^2^ = 0.96).

**Fig 4:**
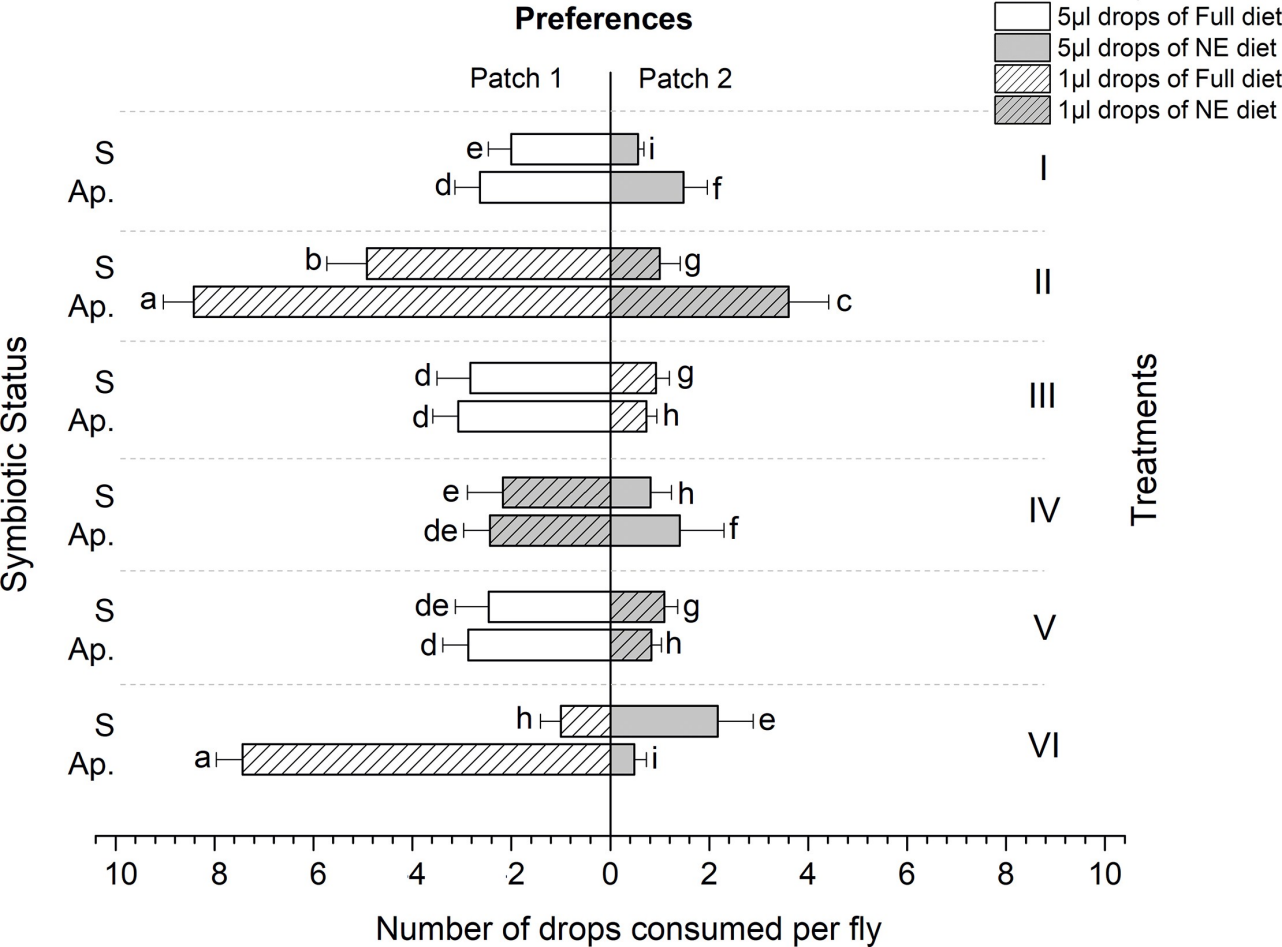
Number of nutritional drops consumed by symbiotic (S) and aposymbiotic (Ap.) *Bactrocera dorsalis* females exposed to two diet patches (full and non-essential amino acids’ diets). Each bar represents the Mean±SE of consumed drops by each fly.

**Fig 5:**
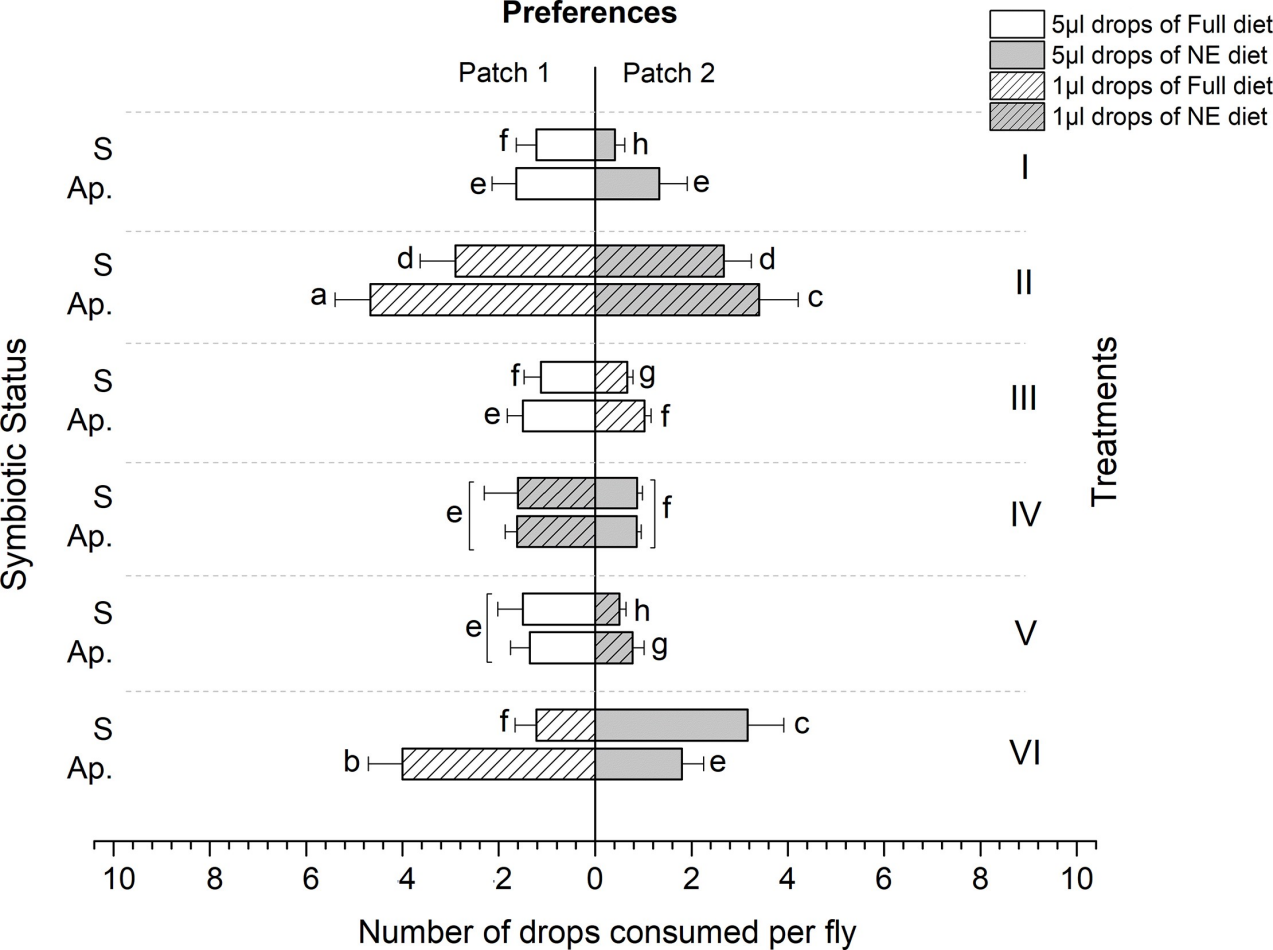
Number of nutritional drops consumed by symbiotic (S) and aposymbiotic (Ap.) *Bactrocera dorsalis* males exposed to two diet patches (full and non-essential amino acids’ diets). Each bar represents the Mean±SE of consumed drops by each fly.

In general, aposymbiotic flies consumed significantly more of food drops than the symbiotic ones irrespective of diet quality and drop size (ANOVA, F = 39.543, df = 1, P < 0.0001, and F = 12.167, df = 1, P = 0.001 in females and males, respectively) (Treatments I, II, V & VI, Figs 4 and 5).

Ingestion of Full diets (high or low volume) from all treatment groups was significantly higher in all tested flies, males and females (F = 64.12, df = 5, P < 0.0001, R^2^ = 0.94, and F = 11.72, df = 5, P < 0.0001, R^2^ = 0.83, respectively) (Figs 4 and 5). Nevertheless, compared to males, females displayed a significant preference toward diets with high reward (full diet, large drops) in unbalanced nutritional environments (F = 41.56, df = 5, P < 0.0001, r^2^ = 0.87) (Figs 4 and 5).

Aposymbiotic flies of both sexes preferentially chose to feed on the Full diet in most treatments, regardless of drop size (except aposymbiotic males in treatment I, who consumed the large Full and NE drops offered to the same extent) (Fig 5). Most importantly, symbiotic condition significantly affected fly feeding behavior in treatment VI. Here, flies were offered many low volume drops of the Full diet, together with few high volume drops of the NE diet. Both male and female aposymbiotic flies were compelled to consume numerous drops of the low volume, Full diet drops, but aposymbiotic male flies ingested higher overall amount of NE diet drops (Fig 5). Interestingly, the actual volume of NE diet (presented in the large drops) consumed by aposymbiotic males was higher than the volume of the Full diet ingested (Fig 5, Treatment VI) (F = 14.22, df = 5, P < 0.0001, R^2^ = 0.49, and F = 5.01, df = 5, P < 0.0001, R^2^ = 0.38, for males and females, respectively) (Figs 4 and 5).

### Allocation of time to feeding

**Fig 6:**
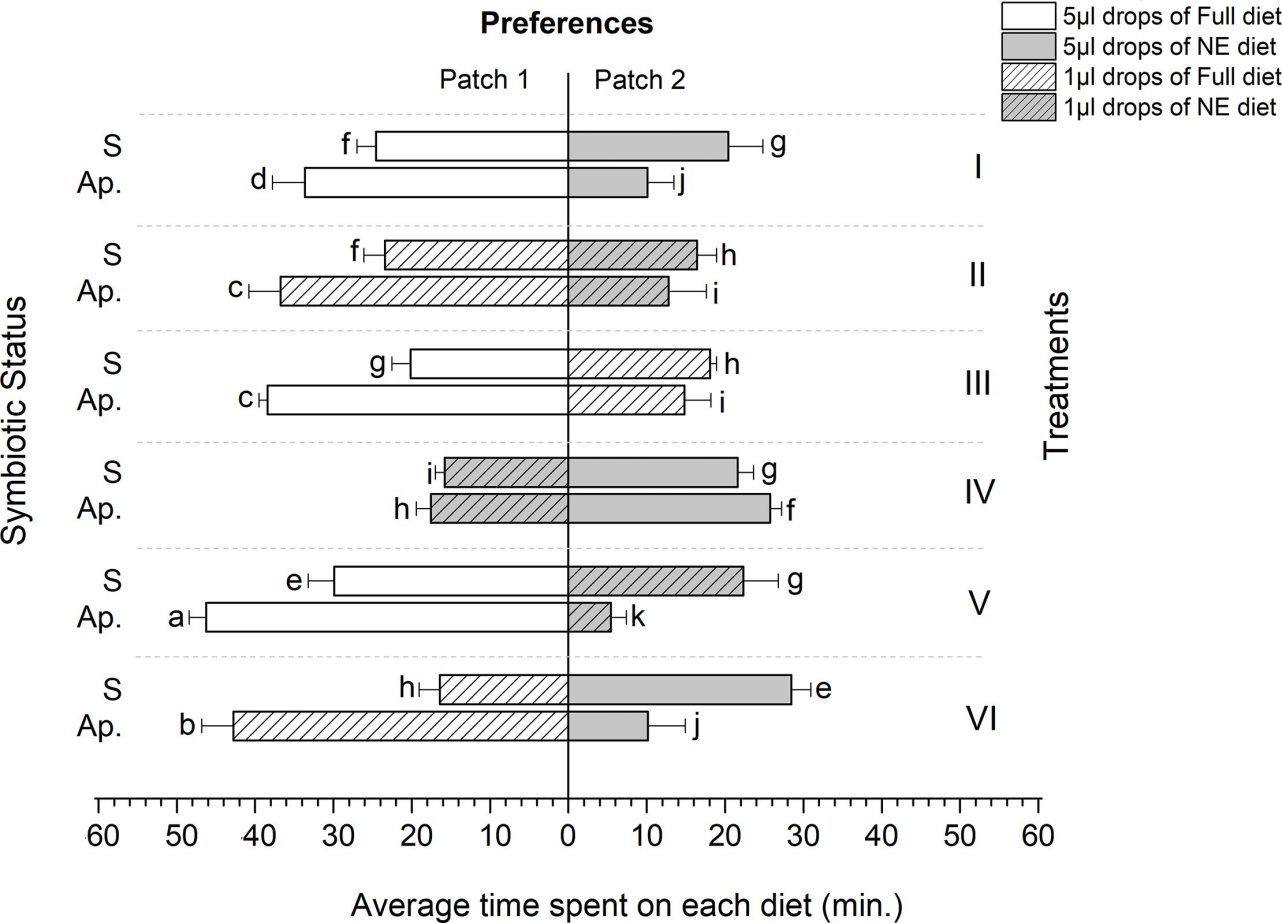
Feeding duration of *Bactrocera dorsalis* females under different foraging environments (Treatments). S: Symbiotic and Ap.: Aposymbiotic. Mean values followed by different letters are statistically different after Tukey HSD Test at P = 0.05.

**Fig 7:**
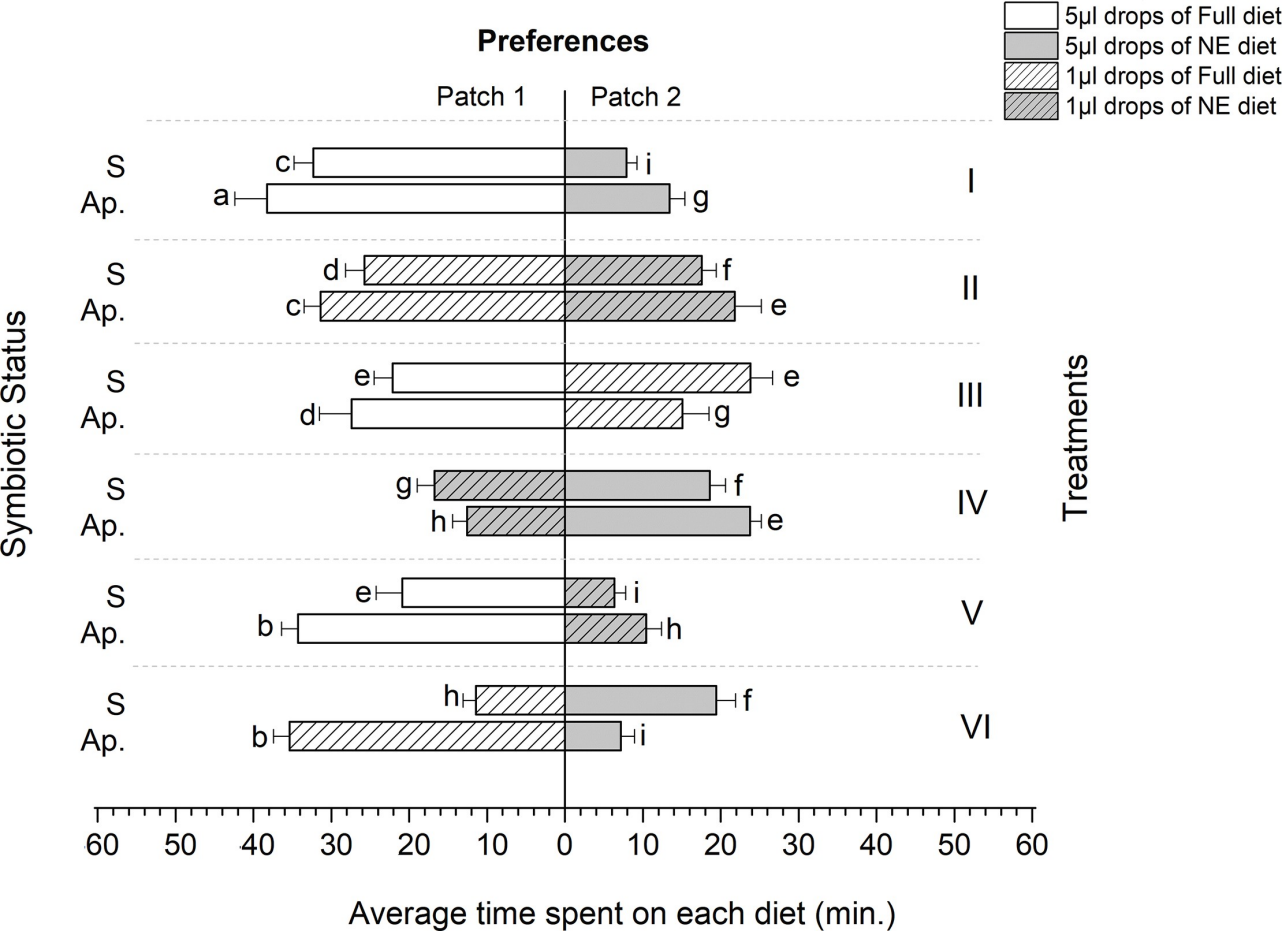
Feeding duration of *Bactrocera dorsalis* males under different foraging environments (Treatments). S: Symbiotic and Ap.: Aposymbiotic. Mean values followed by different letters are statistically different after Tukey HSD Test at P = 0.05.

With the exception of symbiotic females and males in treatment VI, all the experimental flies spent more time foraging on Full diets (Figs 6 and 7). However, the longest time spent on Full diets was recorded in aposymbiotic flies regardless of drop size. Overall, aposymbiotic females spent on average 46.27±2.15 minutes on Full diets compared to 28.43±2.49 minutes by symbiotic females (F = 94.52, df = 5, P = 0.023, R^2^ = 0.96) (Figs 6 and 7). Similarly, aposymbiotic males spent 38.27±4.15 minutes on Full diets, compared to 19.43±2.49 minutes for symbiotic males (F = 33.14, df = 5, P = 0.041, R^2^ = 0.92) (Figs 6 and 7).

A comparison based on drop size also revealed a significantly longer time spent on high volume drops by aposymbiotic flies, except in treatment VI (Figs 6 and 7). Aposymbiotic flies feeding on patches containing NE diets recorded the shortest time spent, with an average time of 5.43±1.95 minutes per patch visited by females compared to 46.27±2.15 minutes when the flies fed on patches containing Full diets. Similarly, aposymbiotic males spent 7.9±1.33 minutes feeding on patches containing NE diets compared to 38.27±4.15 minutes when they fed on patches containing Full diets (Figs 6 and 7).

## Discussion

Foraging entails decision making, whereby each individual must consider trade-offs between energetic and nutrient gain, and the time and risk associated with this activity [2]. Furthermore, when organisms need to ingest nutrients from various food sources, behavioral mechanisms that optimize intake come into play [4]. Gut bacteria have been implicated in this decision making process, both in invertebrates [20,22] and vertebrates [12,43].

In our experiments, when the symbiotic flies were presented a combination of different patch volumes (Treatments III, IV, V, and VI, Fig 1), they preferentially consumed larger drops (Treatments III, V and VI) but when they chose to feed on small drops, they generally consumed a higher number compared to larger drops irrespective of the diet quality presumably to become satiated (Treatment IV). This selective behavior had direct effects on the time spent feeding on a given patch. Our result confirms the abundant previous evidence whereby insects adopt a variety of mechanisms which allow them to optimally regulate the amount of food they ingest when they are confronted with an imbalanced foraging environment, to rapidly reach their nutritional target and limit their exposure [44–46].

The suppression of the microbiome using antibiotics resulted in significant changes of the foraging behavior of both male and female flies. The flies starved for 24 hours prior to bioassays to improve appetitive motivation and allow them to recover from any unintended effects of the antibiotic that could potentially influence behavior. Previous work on medfly [47] and olive fly [28,31] determined that the behavioral changes of the flies stem from the absence of bacteria and not the presence of the antibiotic in the flies system. Accordingly, the overall behavioral changes observed in our experiments can be attributed to the absence of gut bacteria. Aposymbiotic flies responded faster to the diets offered in experimental arenas, spent more time feeding, ingested more drops of food, and were constrained to feed on time consuming patches (containing small drops of food), when these were offered the full complement of amino acids (treatment VI). These findings join a number of recent studies [15–17] in understanding the effect of gut bacteria on different stages of the nexus linking the gut to behavior, and significantly, extend this nexus to patterns of active foraging.

Aposymbiotic flies responded at a higher rate and with shorter latency to the experimental foraging arenas, compared to symbiotic flies (Figs 2 and 3). This suggests that the absence of bacteria in the gut affected the motivational state of these flies, presumably by lowering the response threshold to visual and olfactory stimuli associated with food. In insects, response thresholds to external chemical and visual stimuli are modulated by physiological status [6], which in turn is affected by the presence and composition of the gut microbiome [15,16]. Our results join previous studies in suggesting that the presence or absence of intestinal bacteria can affect behavioral thresholds [7,20,48].

The flies in our experiments, both symbiotic and aposymbiotic, were maintained on a Nitrogen free diet prior to their introduction into the foraging arenas. Previous work on tephritids has established that the gut microbiome is capable of transforming non-essential amino acids (and other intractable sources of Nitrogen), into the building blocks necessary for reproduction and development [26,31,34] (MA and CYN, unpublished data). Accordingly, we predicted that, when presented with a choice between a diet containing only the non-essential amino acids and a diet containing all amino acids, although both flies (symbiotic and aposymbiotic) would behave in a manner consistent with optimal foraging theory, aposymbiotic flies would be constrained to forage preferentially on Full diets, at the cost of significantly extending the amount of time spent foraging. Indeed, the results of our experiment support these predictions. Symbiotic flies significantly spent less time feeding than aposymbiotic flies, and achieved this (energetic and risk averse) saving by feeding on large drops, irrespective of the diet they contained. Conversely, aposymbiotic flies spent more time ingesting food drops, and were compelled to seek drops containing the full diet, even when this choice entailed ingesting a large number of small drops when large (but essential amino acid deficient) drops were available, as in treatment VI (Figs 6 and 7).

We suggest that the next dimension to be explored in this context is a life history one. In monophagous species obligatory gut symbionts enable exploitation of otherwise toxic host plants during the larval stage [28,49], or facultatively enable expansion of the native host range [50]. Empirically, the microbiome of polyphagous tephritids is more varied than that of monophagous tephritid species [23,28,51]. Thus the ability of the microbiome to contribute to the larval phase may come at a cost during the adult phase, when a varied microbiome may be advantageous. In this study we examined the foraging behavior of adult oriental fruit flies, a polyphagous species, with a varied microbiome [24,37,38].

## Conclusion

The results of our study support the emergent paradigm of the effect of gut bacteria on their hosts, affecting gustatory thresholds, feeding behavior and ultimately (as shown here), to patterns of foraging in imbalanced nutritional environments. In future studies, we plan to add a life history dimension to these observations and examine the performance of monophagous flies in similar experimental foraging environments.

## Supporting information

S1 Table Foraging data and statistic models. (XLSX)
